# MiR‐21‐5p Protects Embryonic Growth and Heart Function During Developmental Hypoxia by Dampening HIF Responses and Altering Gene Expression

**DOI:** 10.1002/cph4.70173

**Published:** 2026-05-15

**Authors:** Bernardo J. Krause, Ximena Calle, Ranya V. Kumar, Youguo Niu, Oriana Ramirez‐Herrera, Damariz Gonzalez, Barbara Bernal, Sage G. Ford, Rachael C. Crew, Anna L. K. Cochrane, German A. Arenas, Alex Di Genova, Dino A. Giussani

**Affiliations:** ^1^ Institute of Heath Sciences Universidad O'Higgins O'Higgins Chile; ^2^ Centro UOH de Bioingenieria (CUBI) Universidad O'Higgins O'Higgins Chile; ^3^ Advanced Center of Chronic Diseases (ACCDiS), Facultad de Ciencias Químicas y Farmacéuticas y Facultad de Medicina Universidad de Chile Santiago Chile; ^4^ Department of Physiology, Development & Neuroscience University of Cambridge Cambridge UK; ^5^ Institute of Engineering Sciences Universidad O'Higgins O'Higgins Chile; ^6^ BHF Cambridge Cardiovascular Interdisciplinary Research Centre University of Cambridge Cambridge UK; ^7^ Cambridge Reproduction Interdisciplinary Research Centre University of Cambridge Cambridge UK; ^8^ Department of Obstetrics & Gynaecology University of Cambridge Cambridge UK; ^9^ School of Human Sciences The University of Western Australia Crawley Western Australia Australia; ^10^ Loke Centre for Trophoblast Research University of Cambridge Cambridge UK

**Keywords:** cardiac dysfunction, chronic fetal hypoxia, fetal treatment, miR‐21

## Abstract

Fetal hypoxia is a common complication of pregnancy and increases the cardiovascular risk in offspring, contributing to a significant burden on life‐long health. Preventative strategies against its adverse effects have focused on targeting oxidative stress, but not epigenetic mechanisms. Since miRNA‐21‐5p downregulation contributes to fetal growth restriction (FGR) and cardiovascular impairment, we tested the hypothesis that miR‐21 upregulation during developmental hypoxia is protective. The hypothesis was tested adopting an integrative approach, combining gene expression and transcript variant usage, profiling of neonatal rat cardiomyocytes (NRC) exposed to hypoxia and miR‐21‐5p mimic, and in vivo assessment of the effects of miR‐21‐5p on growth and cardiac function in hypoxic chicken embryos. In NRC, hypoxia resulted in 385 differentially expressed genes (DEG) relative to normoxia (FDR < 0.05; DEG; 142 down‐ and 243 up‐regulated). In vitro miR‐21‐5p improved cell survival, completely dampened hypoxia‐responsive genes, modified transcript isoform usage of key cardiac transcripts (i.e., Sfpq and Lgr4), attenuated apoptotic signaling, and shifted gene networks toward improved Ca^2+^ handling, β‐adrenergic and PI3K‐related pathways. In vivo miR‐21‐5p administration during incubation prevented asymmetric FGR, protected systolic and diastolic function, and normalized cardiac sympathetic dominance in hypoxic chicken embryos. These data suggest that miR‐21‐5p exerts post‐transcriptional control, linking hypoxia sensing to effectors of cardiomyocyte survival and function, as well as protecting against FGR and cardiac dysfunction in development during chronic hypoxia. This work supports the significant translational potential of miRNA‐21 not only as a biomarker, but also as a therapeutic target in high‐risk pregnancy.

## Graphical abstract

miR‐21‐5p protects embryonic growth and heart function during developmental hypoxia by dampening HIF responses and shifting gene networks toward improved cardiomyocyte survival and calcium handling. This work identifies miR‐21‐5p as a promising therapeutic target for preventing fetal growth restriction and cardiovascular impairment in high‐risk pregnancies.

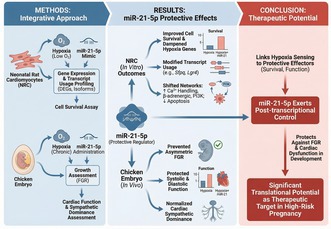

## 1 Introduction

Oxygen levels are crucial in regulating cardiovascular function from the earliest stages of embryonic development and over the longer term (Giussani 2021; Krause 2021). In fetal life, acute hypoxia triggers differential vasomotor responses that redirect blood flow away from the peripheral circulation and toward the developing central nervous system; the so‐called fetal brain‐sparing effect (Giussani et al. 1994; Thakor and Giussani 2009; Giussani 2021). While short‐term cardiovascular responses to acute hypoxia are designed to be protective, sustained cardiovascular responses to chronic hypoxia may become maladaptive, claiming many trade‐offs that can increase disease risk vulnerability. Chronic fetal hypoxia promotes sympathetic dominance in the fetal heart and vasculature (Ruijtenbeek et al. 2000; Niu et al. 2018; Darby et al. 2021), endothelial dysfunction (Giussani and Davidge 2013; Herrera et al. 2017; Paz et al. 2019; Krause et al. 2024), and delays the transition from proliferative to terminally differentiated cardiomyocytes, remodeling myocardial structure (Patey et al. 2025; Popazova et al. 2025). Therefore, chronic fetal hypoxia is known to trigger a fetal origin of cardiovascular dysfunction and program an increased cardiovascular risk in the adult offspring (Giussani and Davidge 2013; Giussani 2021).

These significant effects of chronic hypoxia on the cardiovascular susceptibility of the offspring have prompted researchers to investigate potential interventions, focusing primarily on antioxidant therapies (Botting‐Lawford et al. 2025). However, adaptations occurring in cardiovascular structure and function, including those elicited by hypoxia, are also strongly influenced by epigenetic mechanisms that can change gene expression without altering the underlying DNA sequence (Krause 2021; Krause et al. 2024). These mechanisms may alter gene expression without altering the underlying DNA sequence, involve DNA methylation, histone post‐translational modifications, ATP‐dependent chromatin modifications, and non‐coding RNAs (Cavalli and Heard 2019). For instance, miRNAs abundantly expressed in the vasculature, such as miR‐21, play a crucial role in the endothelial responses to hypoxia and disturbed blood flow (Marin et al. 2013; Gonzalez‐Candia et al. 2024). We have recently demonstrated that miR‐21 expression is down‐regulated by acute hypoxia in human fetuses exposed to chronic hypoxia and contributes to regulating several vasoactive genes in fetal and umbilical arterial endothelial cells (Penaloza et al. 2020; Vega‐Tapia et al. 2021). Similarly, maternal cigarette smoking during pregnancy is associated with the downregulation of miR‐21 in the placenta (Maccani et al. 2011) and low placental miR‐21 expression predicts low birth weight (Maccani et al. 2010; Kochhar et al. 2022; Sekovanic et al. 2022). However, whether miR‐21 upregulation during developmental hypoxia is protective of fetal growth and cardiovascular function has never been investigated. Therefore, the aim of this study was to test the hypothesis that miR‐21 upregulation during developmental hypoxia is protective by combining gene expression and transcript variant usage, profiling of neonatal rat cardiomyocytes (NRC) exposed hypoxia and miR‐21‐5p mimic, and in vivo assessment of the effects of miR‐21‐5p on growth and cardiac function in hypoxic chicken embryos.

## 2 Materials and Methods

### 2.1 Ethics Statement

Studies in chicken embryos were performed under the UK Animals (Scientific Procedures) Act 1986 and were approved by the Ethical Review Board of the University of Cambridge. Studies in neonatal rat cardiomyocytes were approved by the Ethical Review Board of the University of Chile (protocol number 200351 CYQ‐UCH). All experiments were designed and reported concerning the ARRIVE guidelines (Percie du Sert et al. 2020).

### 2.2 In Vivo Chronic Hypoxia

Fertilized Bovans Brown eggs (*Gallus gallus domesticus*) were purchased from Medeggs (Henry Stewart & Co., Norfolk, UK), weighed, and incubated under normoxic (21% O_2_) or hypoxic (14% ± 0.5% O_2_) conditions from day 1. Aside from oxygen levels, both groups were exposed to equal environmental conditions, consisting of 37.9°C ambient temperature, 45% humidity, 12:12 h light/dark cycle, and automatic rotation every hour in an incubator (Masalles incubator Mod‐75A, equipped with electronic servo‐controlled humidity cool steam injection system HS‐Auto‐3.5L; Masalles, Barcelona, Spain). The oxygen levels, humidity, and temperature inside the incubators were continuously monitored (DD103 DrDAQ Oxygen Sensor, Pico Technology, St. Neots, UK) (Krause et al. 2024).

Chicken embryos incubating under normoxia or chronic hypoxia were treated with agomiR‐21‐5p (1 μg/embryo every 2 days, Sigma‐Aldrich, Dorset, UK) or vehicle (100 μL saline) from Day 13 to 19 of the 21‐day incubation period. Treatment was administered topically onto the chorioallantoic membrane daily via a 1‐mm hole in the eggshell through the air cell. The hole in the eggshell was covered with a small piece of tape at all other times, and all treatment procedures were performed under sterile conditions. Treatment of hypoxic chicken embryos occurred in a side chamber attached to the hypoxic incubator, which was maintained at the same level of oxygenation so that treatment occurred without losing the hypoxic exposure (Krause et al. 2024). In human pregnancy complicated by hypoxia, the ideal therapy would need to rescue fetal origins of cardiovascular disease following diagnosis of FGR. In current obstetric practice, pregnant women identified at risk of FGR are referred for uterine artery Doppler and serial ultrasound measurement for fetal size between 20 and 24 weeks of gestation. Therefore, treatment with miRNA of pregnancy complicated by chronic hypoxia would have to start from 0.6 of gestation following earlier FGR diagnosis. Therefore, the dosing protocol, administration and concentration were derived from previous studies investigating the effects of exogenous miRNA during development in chicken embryos at concentrations that do not result in non‐specific targeting (McGlinn et al. 2009; Wittig et al. 2019). Additionally, the treatment period (starting at Day 13) was designed to model the human situation where treatment would have to start from 06 of gestation following diagnosis of FGR in human pregnancy [reviewed in (Itani et al. 2018)]. Since there is complete species conservation of the miR‐21‐5p sequence among chickens, rats and humans, and to increase the translational value of the intervention, mature human miR‐21‐5p (hsa‐miR‐21‐5p) was used. On Day 19 of the 21‐day incubation period, chicken embryos underwent euthanasia by cervical spinal transection. The embryo was removed from the shell and blotted with paper towel. The chick body weight and crown‐rump length (CRL) were recorded, and the brain was dissected and weighed, as described previously (Krause et al. 2024).

### 2.3 Ex Vivo Cardiac Function

After chicken embryo body weight was recorded, the heart was rapidly excised and immediately placed into ice‐cold Krebs–Henseleit bicarbonate (KHB) buffer (120 mM NaCl, 4.7 mM KCl, 1.2 mM MgSO_2_·7H_2_O, 1.2 mM KH_2_PO_4_, 25 mM NaHCO_3_, 10 mM glucose, and 1.3 mM CaCl_2_·2H_2_O). The heart was cannulated via the aorta using a canula made from a 19‐G needle and perfused via the coronary arteries at a constant pressure of 40 cmH_2_O. A small flexible nonelastic balloon was inserted into the left ventricle (LV) through the left atrium (LA). The balloon was then filled with distilled water and attached to a rigid, distilled water‐filled catheter connected to a calibrated pressure transducer (Argon Medical Devices, Plano, TX, USA). The volume of the balloon was adjusted to around 30 μL by injection of distilled water with a 100 μL Hamilton syringe to get a recording of LV end diastolic pressure (LVEDP) between 5 and 10 mmHg. Recirculating KHB was filtered through a 5 μm cellulose nitrate filter (Millipore, Bedford, MA, USA) and gassed with O_2_:CO_2_ (95:5%) at 40°C. After an initial stabilization period of 15 min, basal measurements of heart rate (HR), LV systolic pressure (LVSP), and LVEDP were recorded. Baseline LV developed pressure (LVDP) was calculated as LVSP‐LVEDP. The maximum (dP/dt_max_) and minimum (dP/dt_min_) first derivatives of LV pressure were calculated automatically using the M‐PAQ data acquisition system (Maastricht Programmable AcQusition System, Maastricht, Netherlands).

To examine stimulated cardiac function, the cardiac chronotropic and inotropic responsiveness to the muscarinic receptor agonist carbachol (carbamylcholine chloride; Sigma‐Aldrich Co Ltd., Poole, United Kingdom; range, 10^−8^–10^−6^ mol/L) and the β_1_‐adrenoreceptor agonist isoprenaline ([‐]‐Isoproterenol [+]‐bitartrate salt; Sigma‐Aldrich Ltd., Poole, United Kingdom; range, 10^−9^–10^−7^ mol/L) were investigated. Carbachol and isoprenaline were dissolved in KHB and administered into the heart via the compliance chamber of the Langendorff apparatus. The heart was perfused in a non‐recirculating way to avoid accumulation of carbachol or isoprenaline within the system. Recovery time (ranging from 5 to 15 min) was allowed between each bolus to allow HR and LVDP to stabilize to baseline values before the administration of the next bolus. The HR and LVDP responses were expressed as percentage change from the baseline, and then, the ratio of the maximal HR (chronotropic) and LVDP (inotropic) responses to isoprenaline and to carbachol was calculated (Itani et al. 2016; Hess et al. 2020).

### 2.4 Assessment of Effects on Embryo Growth

Considering the potential broad effects of the treatments applied administered topically onto the chorioallantoic membrane, growth efficiency and partition of resources were determined as previously described (Giussani et al. 2007). Briefly, growth efficiency was calculated as the ratio of the “measured fetal weight at Day 19” and the product of “the egg weight at Day 19” minus “the weight of the egg once the embryo had been removed at Day 19.” To calculate the partitioning of the resources, both the “measured fetal weight at Day 19” and “egg weight at Day 19–the measured fetal weight at Day 19” were expressed as a percentage of the egg weight at Day 19 and plotted in histogram format.

### 2.5 Primary Culture

Neonatal ventricular myocytes were prepared from hearts of 1–3‐day‐old Harlan Sprague Dawley rats as described previously (Tao et al. 2018). Selection of this tissue as an in vitro model was based on the comparative developmental stage that displays neonatal rodent cardiac cells with fetal human and chicken counterparts (Itani et al. 2018), enabling a comparison of the early effects of the treatment applied and the in vivo intervention studied. Briefly, ventricles were trisected, pooled, and myocytes dissociated in a solution of collagenase and pancreatin. After enzymatic dissociation, the cells were selectively enriched for cardiac myocytes by being pre‐plated in DMEM/M199 (4:1) containing 10% (v/v) horse serum, 5% (v/v) heated‐inactivated fetal calf serum, penicillin, and streptomycin (100 units/mL). The myocytes, plated at a final density of 1.0–1.4 × 10^3^/mm^2^ on gelatin‐precoated 35‐mm or 60‐mm dishes, respectively, were confluent and spontaneously beating after 18 h.

### 2.6 In Vitro miR‐21‐5p Transfection

MicroRNA transfection was performed as previously described (Penaloza et al. 2020). Briefly, cultured cells were transfected with 30 nM mimic miR‐21‐5p (Thermofisher) or control scramble, using LipofectamineTM RNAiMAX reagent (Invitrogen), by incubating in transfection medium (Opti‐MEM reduced serum medium without phenol red) for 6 h and then exposed to hypoxia (1% oxygen) or normoxia for 6 h.

### 2.7 Detection of Apoptotic Cardiomyocytes by the TUNEL Assay

Apoptotic cells were detected by the TUNEL method using an in situ detection kit (Promega) according to the manufacturer protocol. Briefly, cells were cultured on circular coverslips coated with 2% gelatin, fixed in 4% paraformaldehyde for 25 min at 4°C, and then washed in PBS three times for 5 min each. The fixed cells were permeabilized with 0.2% Triton X‐100 in PBS for 5 min and then incubated with fluorescein‐labeled dUTP for 60 min at 37°C to detect the free 3′ hydroxyl fragmented DNA ends. After washing in PBS, apoptotic nuclei were visualized by immunofluorescence after all nuclei were stained with DAPI.

### 2.8 Transcriptional Profiling

Transcript profiling was performed on cardiomyocyte whole RNA extracts using long‐read sequencing (Oxford Nanopore Technology; ONT) following the protocols suggested by the provider (available at: https://community.nanoporetech.com/docs/prepare/library_prep_protocols/pcr‐cdna‐barcoding‐kit‐sqk‐pcb111‐24). Briefly, cardiomyocytes were lysed and 200 ng of RNA from each sample was then reverse transcribed for library preparation using the PCR‐cDNA Barcoding kit (SQK‐PCB109). Barcoded cDNA from 16 samples (4 biological replicates from each condition—normoxia, normoxia treated with miR21, hypoxia, and hypoxia treated with miR21) was pooled and loaded into two PromethION Flow Cells for sequencing for 72 h.

Nanopore cDNA raw reads were processed with the nf‐core/nanoseq pipeline (v3.1.0; Nextflow v23.10, “kutral” profile) to obtain expression‐ready counts. Briefly, FastQC (v0.12.1) first assessed base‐called FASTQ files for per‐base quality and adapter contamination. NanoLyse (v1.3) then removed λ‐phage and other spike‐in controls, after which NanoPlot (v1.42) summarized read length, quality, and yield for visual inspection. Barcodes were trimmed (‐‐trim_barcode true) from SQK‐PCS111 libraries, retaining reads with qcat scores ≥ 20, and the cleaned reads were aligned to *Rattus norvegicus* (mRatBN7.2.110) using minimap2 (v2.30, ‐‐preset cdna), with demultiplexing skipped because samples were already split (‐‐skip_demultiplexing true). Finally, bambu (v3.8) quantified known and novel isoforms, producing gene‐ and transcript‐level count matrices. Fusion detection and RNA‐modification modules were disabled (‐‐skip_fusion_analysis true; ‐‐skip_modification_analysis true) to streamline runtime; default parameters were used otherwise.

Differentially expressed genes (DEGs) were identified from pair‐wise comparisons of the four conditions by retaining transcripts whose absolute fold‐change exceeded 1.2 and whose Benjamini‐Hochberg‐adjusted *p*‐value was < 0.05. Exon‐level differential usage was evaluated in parallel with DEXSeq (v1.55), applying the same statistical thresholds to call significantly regulated exons.

### 2.9 Transcriptional Meta‐Analysis

Datasets from fetal hearts of mice exposed to in vivo hypoxia (GSE114532) were analyzed using the Galaxy platform (https://usegalaxy.org) and the R package TCC (https://bioconductor.org/packages/release/bioc/html/TCC.html). Briefly, raw sequencing data were first imported into Galaxy, adapter sequences were trimmed using Trim Galore, and quality control was assessed before and after trimming using FastQC. High‐quality reads were aligned to the *Mus musculus* (mm39) reference genome using HISAT2. Aligned reads (in BAM format) were quantified using featureCounts, which counts reads mapping to genomic features based on the provided gene annotation file. Differential expression analysis was performed in TCC using the count matrix generated by featureCounts was used as input for DESeq2, a statistical package for identifying DEGs in RNA‐seq data. Datasets from hearts of chicken embryos exposed to hypoxia (GSE12675) were analyzed using the Transcriptome Analysis Console 4.0.1 (ThermoFischer Scientific) as previously described (Vega‐Tapia et al. 2021). Samples were grouped according to experimental conditions provided in the metadata, and DEGs were determined based by retaining transcripts whose absolute fold‐change exceeded 1.2 and whose Benjamini‐Hochberg‐adjusted *p*‐value was < 0.05.

### 2.10 Functional Enrichment

DEG lists were further assessed for functional enrichment using DEVEA shiny app (freely accessible at https://shiny.imib.es/devea; source code available on the GitHub repository https://github.com/MiriamRiquelmeP/DEVEA) (Riquelme‐Perez et al. 2022). The analysis considered fold‐change values and adjusted significance to determine enrichment, and results were filtered to cardiovascular‐ and cell viability‐related terms to avoid over‐representation of non‐specific pathways and processes. Gene ontology biological process plotting was restricted to top 20 terms (based on lowest FDR), and ontology levels 4–5 to avoid overrepresentation of terms and asymmetries in gene ratio levels. Enrichment plots were generated in R using GraphBio (https://github.com/databio2022/GraphBio), and UpSetR and enrichplot packages.

### 2.11 Statistics

Values are expressed as the mean ± SEM. For the myography experiments, LabChart was used for data acquisition and analysis (Labchart 6.0, Powerlab 8/30; AD Instruments, Chalgrove, UK). Concentration‐response curves were determined using an agonist‐response best‐fit line in Prism 10.0; GraphPad Software. For all data, comparisons were made using one or two‐way ANOVA with and without repeated measures, as appropriate. Relationships between variables were determined using the Pearson correlation. The Mann–Whitney test was used for continuous variables and the chi‐square for frequency comparisons. For all tests, differences were considered statistically significant when *p* < 0.05 (Prism 10.0; GraphPad Software).

## 3 Results

### 3.1 MiR‐21‐5p Attenuates Hypoxia‐Induced Apoptosis and Transcriptomic Remodeling in Neonatal Rat Cardiomyocytes

**FIGURE 1:**
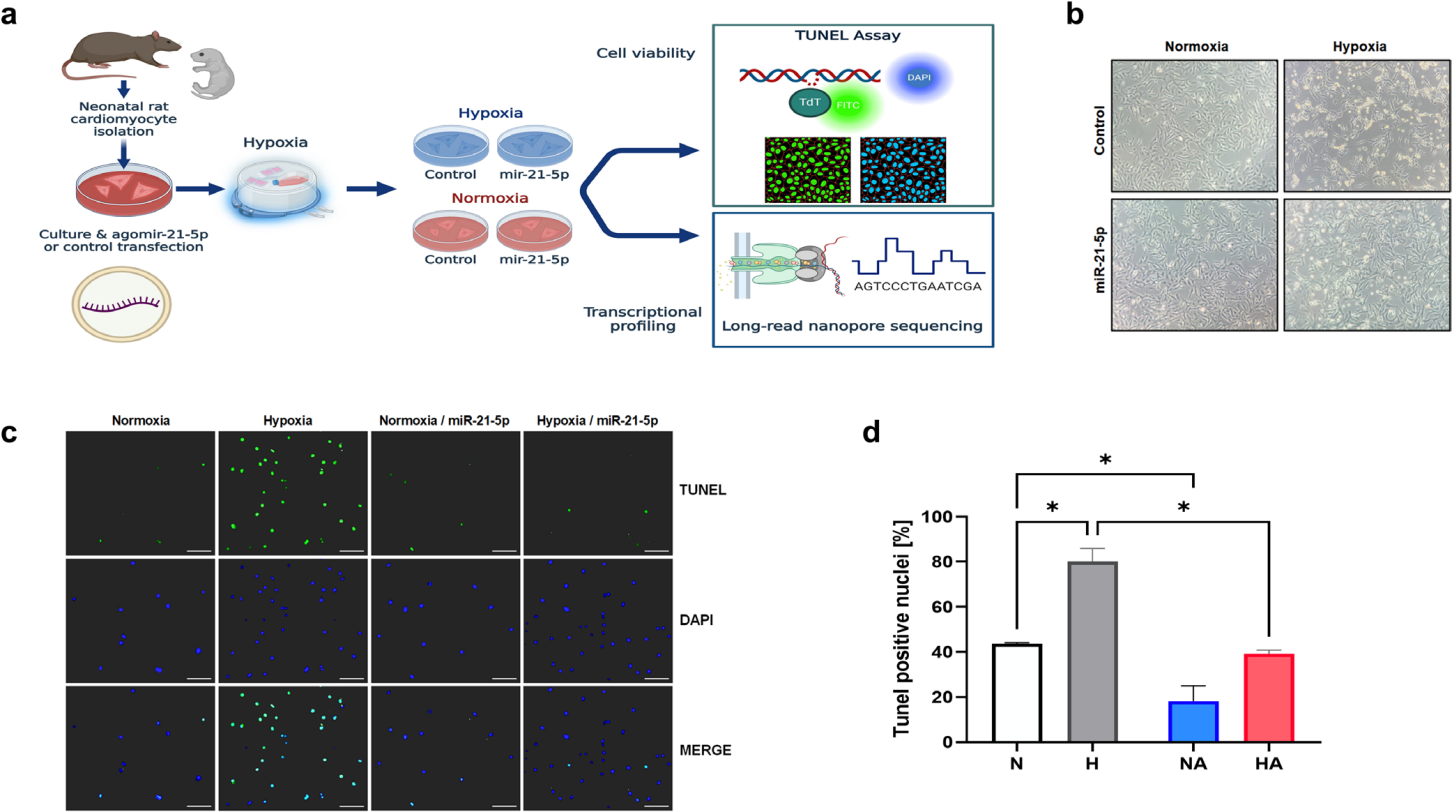
miRNA‐21‐5p improves cell survival in cardiomyocytes exposed to in vitro hypoxia. (a) Experimental design for assessing the role of miR‐21‐5p on the hypoxic response in neonatal rat cardiomyocytes (NRC). (b) Representative micrographs of cultured NRC after incubation under hypoxia for 6 h. (c, d), Tunnel staining in NRC cultured under normoxia (N, open bar, *n* = 4) hypoxia (H, gray bar, *n* = 4), normoxia with miR‐21‐5p (NA, blue bar, *n* = 4), and hypoxia with miR‐21‐5p (HA, red bar, *n* = 4). Experimental design figure in a was created with BioRender. Values expressed as mean ± SEM. *Two‐way ANOVA with FDR post hoc comparison.

To address this, we studied the transcriptional response elicited by hypoxia in neonatal rat cardiomyocytes (NRC) and the regulation of this hypoxia response by miR‐21‐5p. Cultured NCR were pretreated with miR‐21‐5p or mock, submitted to hypoxia for 6 h, after which cell viability and transcriptional profiling (long‐read Nanopore sequencing) were assessed (Figure 1a). Cells exposed to hypoxia showed increased levels of TUNEL‐positive nuclei compared to normoxic NRC, and this effect was reversed by pre‐treatment with miR‐21‐5p (Figure 1b,c).

**FIGURE 2:**
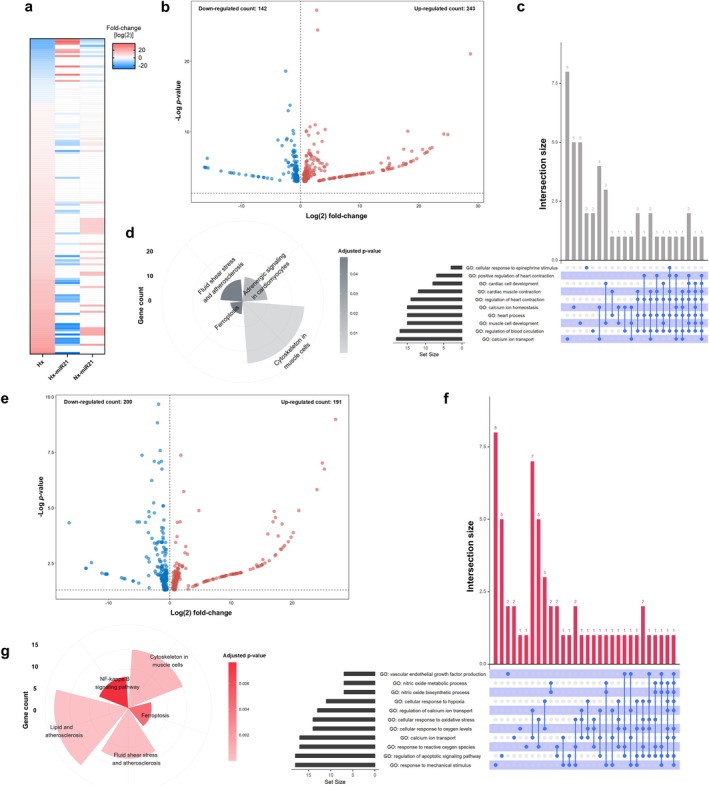
miRNA‐21‐5p prevents the transcriptional effects of hypoxia in cardiomyocytes. (a) Heatmap of differentially expressed genes relative to normoxia in NRC exposed to hypoxia (first lane), hypoxia with miR‐21‐5p (second lane), and normoxia with miR‐21‐5p (third lane), ordered from the lowest (top) to the highest (bottom) according to hypoxia. Fold changes are ordered from down‐ to up‐regulated according to changes in hypoxia, with blueish denoting down‐regulation, white for no changes, and reddish for up‐regulation. Volcano plots showing DEG relative to normoxia in NRC exposed to hypoxia (b) or hypoxia with miR‐21‐5p (e). UpSet plot showing the overlap among enriched genes (horizontal bars represent the number of genes in each gene set, while the vertical bars show the size of the overlap between sets, and connected dots below the vertical bars indicate which gene sets are included in each intersection) in NRC exposed to hypoxia (c) or hypoxia with miR‐21‐5p (f). Polar plot displaying Kegg pathways enriched in NRC exposed to hypoxia (d) or hypoxia with miR‐21‐5p (g).

At the transcriptional level, hypoxia resulted in 385 differentially expressed genes (DEG) relative to normoxia (FDR < 0.05; DEG; 142 down‐ and 243 up‐regulated; Figure 2a,b). Specific pathways that were enriched included those involved in cardiac function, calcium homeostasis, adrenergic signaling, and apoptosis (Figure 2c,d). To further explore these transcriptional responses to hypoxia, enriched pathways were further validated by comparing data from mice (GSE114532) and chickens (GSE12675) exposed in vivo to hypoxia during early development. This revealed several similarities in KEGG pathways and gene ontology biological processes at the transcriptional level which were differentially regulated by hypoxia in cardiac tissue, either in vivo or in vitro (Figure S1). This data strongly suggests that early and long‐term responses to fetal hypoxia result in the up‐regulation of transcriptional pathways promoting an increased adrenergic response, diminished cell survival, and cardiomyopathy.

**FIGURE 3:**
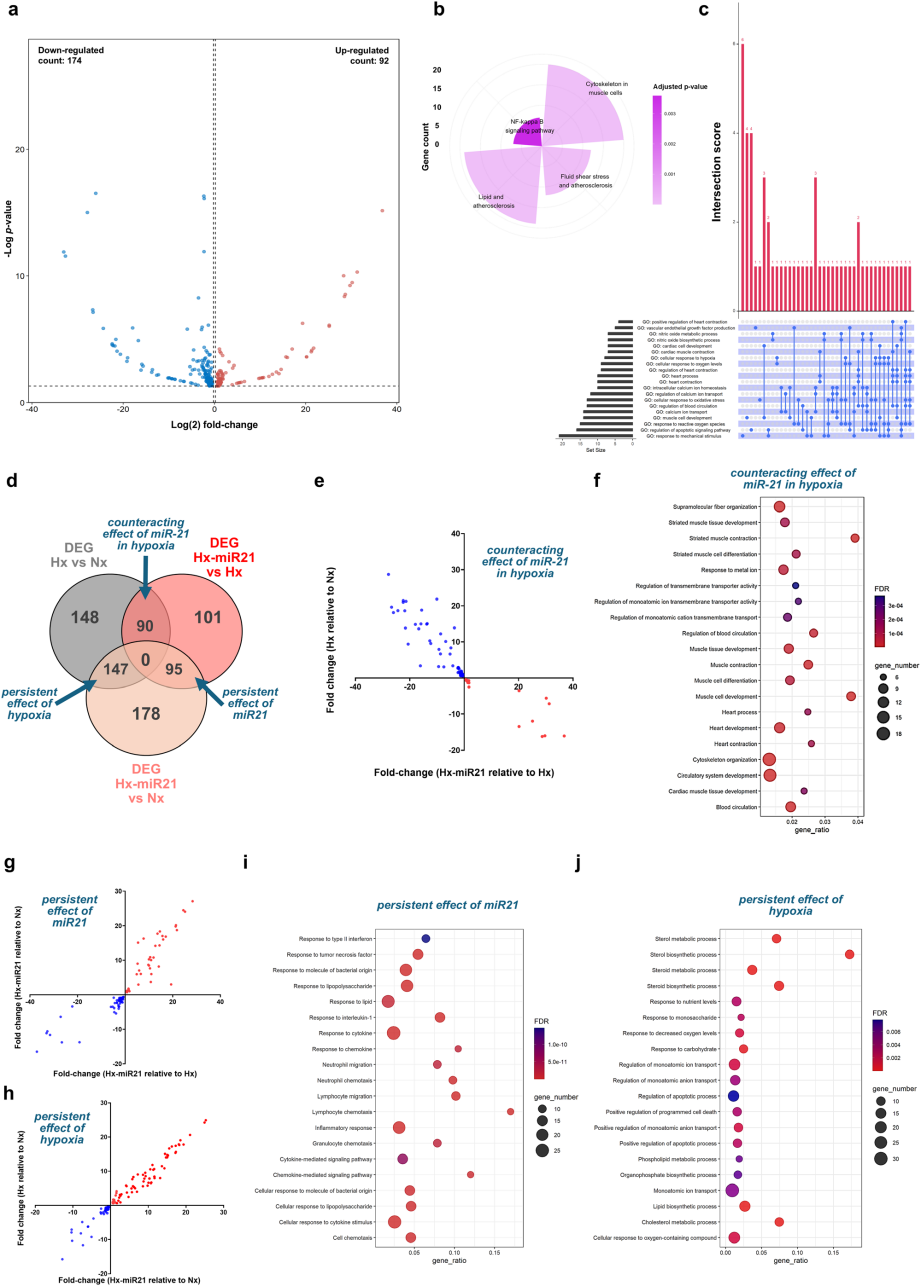
miRNA‐21‐5p dampens the transcriptional effects of hypoxia i**n** cardiomyocytes. (a) Volcano plots showing DEG relative to hypoxia in NRC exposed to hypoxia with miR‐21‐5p. (b) Polar plot displaying Kegg pathways enriched in NRC exposed to hypoxia in the presence of miR‐21‐5p, (c) and UpSet plot showing the overlap among enriched genes (horizontal bars represent the number of genes in each gene set, while the vertical bars show the size of the between sets, and connected dots below the vertical bars indicate which gene sets are included in each intersection). (d) Venn‐diagram integrating DEG found in hypoxic NRC versus normoxia, hypoxic miR‐21‐5p‐treated NRC versus hypoxia, and hypoxic miR‐21‐5p‐treated NRC versus normoxia. Comparisons of the fold changes between among common DEG found in the different conditions compared (e, g, and h). Dot plots of enriched biological process in DEG in the intersections of the Venn‐diagram in d (f, i, and j).

Conversely, pre‐treatment with miR‐21‐5p resulted in 391 DEG (Figure 2e) relative to normoxia, with 200 down‐ and 191 up‐regulated transcripts, enriched in pathways involved in cardioprotection, apoptosis, response to hypoxia, and immune regulation (Figure 2f,g). Analysis of the effects of miR‐21‐5p in hypoxia showed 266 DEG relative to untreated hypoxic NRC, with 174 down‐ and 92 up‐regulated transcripts (Figure 3a), with partial overlap in enriched pathways observed under hypoxia (relative to normoxia) (Figure 3b,c). To isolate the effects of miR‐21‐5p and hypoxia, functional enrichments were performed on DEG found in the intersections of hypoxia (relative to normoxia) and hypoxic NRC treated with miR‐21‐5p (i.e., counteracting effects of miR‐21), hypoxic NRC treated with miR‐21‐5p relative to normoxia and hypoxia (i.e., persistent effect of miR21), and hypoxic NRC relative to normoxia with or without miR‐21‐5p treatment (i.e., persistent effect of hypoxia) (Figure 3d). Counteracting effect of miR‐21 in hypoxia included 90 DEG with a complete inverse regulation (Figure 3e), characterized by enrichment in biological processes related to cardiac function and development (Figure 3f). Persistent effect of miR‐21 included 95 DEG, similarly regulated between the conditions compared (Figure 3g), enriched in immune‐related biological processes (Figure 3i). Conversely, persistent effect of hypoxia included 147 DEG with a comparable regulation between the conditions assessed (Figure 3h), enriched mainly in metabolic biological processes (Figure 3j).

### 3.2 Regulation of Hypoxia Responses by miR‐21‐5p at the Gene and Transcript‐Isoform Level

**FIGURE 4:**
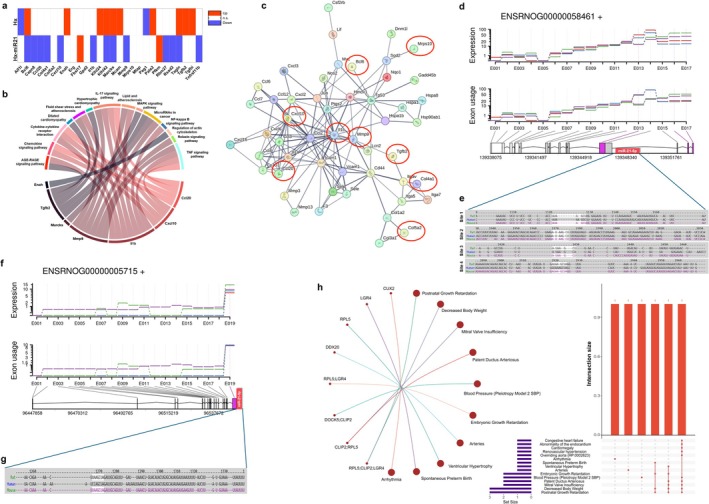
Transcriptional regulation by miR‐21‐5p occurs at gene and transcript levels. (a) miR‐21‐5p targets differentially regulated in NRC exposed to hypoxia and hypoxia with miR‐21‐5p, with blue denoting down‐regulation, white for no changes, and red for up‐regulation. Interaction of miR‐21‐5p targets differentially expressed with enriched Kegg pathways (b) and other DEG (c, miR‐21 target denoted by red circles) occurring in NRC exposed to hypoxia and hypoxia with miR‐21‐5p. Genes with Differential Transcript Usage (DTU) (d—Sfpq, f—Lgr4). The graph illustrates the expression levels (top panel) and relative usage of exons (lower panel) across different transcript isoforms, with colors denoting each trait for control (green lines), hypoxia (red lines), normoxia with miR‐21‐5p (purple lines) and hypoxia with miR‐21‐5p (blue lines). Seed sequences for miR‐21‐5p for Sfpq (e) and Lgr4 (g) and their conservation in the transcript sequences of rats, humans, and mice. (h) Network plot illustrating the enriched pathways and their interconnections, and UpSet plot showing the overlap among enriched terms, for genes with DTU showing their impact on cardiac function and perinatal growth pathways in NRC exposed to hypoxia or hypoxia with miR‐21‐5p.

Using Mirtarbase and Targetscan databases in humans, mice, and rats, when comparing hypoxia, hypoxia with miR‐21‐5p, and normoxia with miR‐21‐5p profiles, we identified 28 DEG reported as miR‐21‐5p targets, of which 23 were differentially regulated between hypoxia and hypoxia with miR‐21‐5p, with a prominent down‐regulation of them in the presence of miR‐21‐5p (Figure 4a). Transcriptional changes in response to miR‐21‐5p under normoxia were subtle compared to hypoxic conditions (Figure S2). Nevertheless, the differential regulation of miR‐21‐5p targets was also associated with commonly enriched KEGG pathways found in both hypoxia and hypoxia with miR‐21‐5p NRC (Figure 4b). Moreover, 9 miR‐21‐5p target DEG showed strong functional interactions with 51 DEG (about 14% of total) occurring in both hypoxia and hypoxia with miR‐21‐5p (Figure 4c), supporting the crucial role of these targets in the protection against hypoxia. Additionally, we performed a Differential Transcript Usage (DTU) analysis to delve into variations in the usage of transcript isoforms across the diverse conditions assessed. This method offers insight into post‐transcriptional regulation and alternative splicing events, often missed by gene‐level differential expression analyses. This assessment is particularly effective using Nanopore sequencing data, especially when examining complex splicing patterns or unannotated regions. We found 11 transcripts that showed differences concerning their exon expression and usage, including Clip2, Cux2, Ddx20, Dock5, Gab2, Lgr4, Myo19, Rps9, Rpl5, Sfpq, and Utp6. Of interest, Sfpq showed a differential usage of the exon 14, whose 3′ UTR region harbored four different binding sites for miR‐21‐5p (Figure 4d), with variable conservation among humans, mice, and rats (Figure 4e). A similar pattern was observed in the 3′ UTR of Lgr4 (Figure 4f,g), however, the miR‐21‐5p binding region was not associated with differential usage. Despite the reduced number of genes with DTU, they showed enrichment in pathways related to cardiac function and perinatal growth (Figure 4h).

### 3.3 In Vivo miR‐21‐5p Administration Preserves Embryonic Growth During Developmental Hypoxia

**FIGURE 5:**
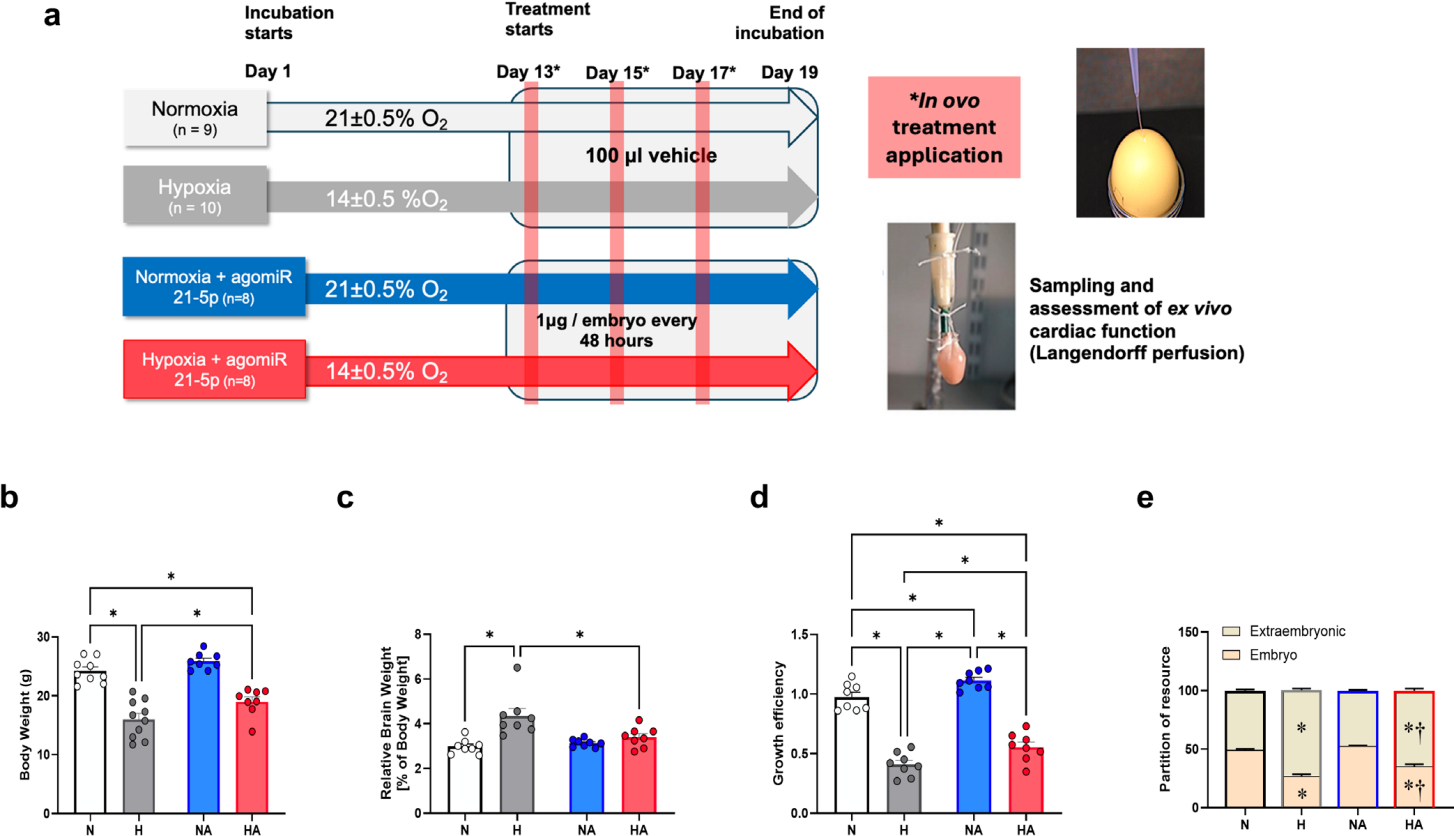
In vivo miR‐21‐5p administration protects growth during developmental hypoxia in the chicken embryo. (a) Experimental design for determining the role of miR‐21‐5p on growth and cardiac function during chronic developmental hypoxia using a chicken embryo model. Hypoxic groups were exposed since Day 1 of incubation to low oxygen levels, meanwhile treatments were applied since Day 13 of incubation. Bar plots showing (b) body weight; (c) relative brain‐to‐body weight ratio; (d) growth efficiency; (e) partition of resource. Groups are normoxia (N, open bar, *n* = 9) hypoxia (H, gray bar, *n* = 10), normoxia with miR‐21‐5p (NA, blue bar, *n* = 8), and hypoxia with miR‐21‐5p (HA, red bar, *n* = 8). Statistical differences are (*p* < 0.05), *Two‐way ANOVA with FDR post hoc comparison (* vs. normoxia, † vs. hypoxia in stacked bars).

Hypoxic incubation of fertilized eggs promoted growth restriction with evidence of brain sparing in the chicken embryo by Day 19 of incubation (term = 21 days), and that treatment with synthetic miR‐21‐5p (agomiR) of hypoxic incubations from Day 13 to 19 prevented the asymmetric growth restriction (Figure 5b,c). Further characterization of the effects of miR‐21‐5p treatment showed a partial improvement in growth efficiency (Figure 5d) and partition of resource (Figure 5e) in hypoxic embryos treated with miR‐21‐5p, as well as higher growth efficiency in normoxic embryos treated with the agomiR.

### 3.4 MiR‐21‐5p Prevents Hypoxia‐Induced Ventricular Dysfunction and the Shifting Toward Sympathetic Dominance

**FIGURE 6:**
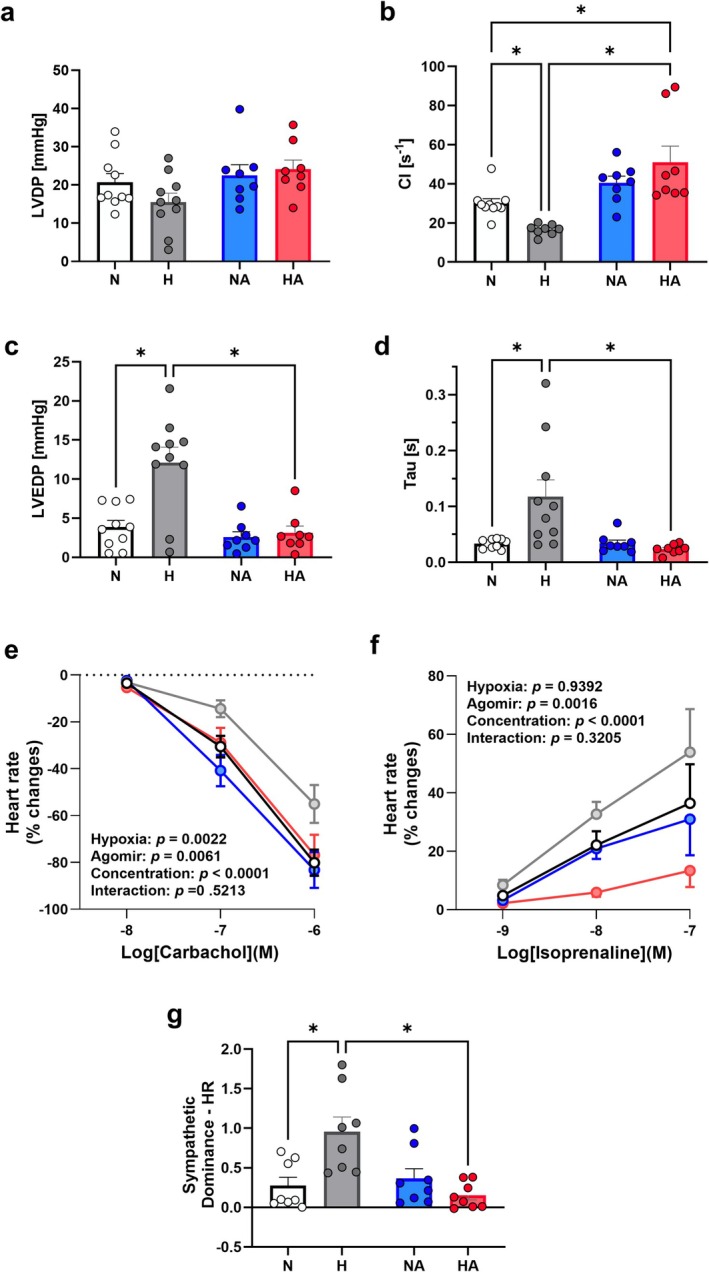
In vivo miR‐21‐5p administration protects cardiac function during developmental hypoxia in the chicken embryo. (a) Left ventricular developed pressure (LVDP); (b) contractility index (CI); (c) left ventricular end‐diastolic pressure (LVEDP); (d) time constant of isovolumic relaxation (Tau); (e) heart rate response to increasing doses of carbachol; (f) heart rate response to increasing doses of isoprenaline; (g) ratio of the maximal response in heart rate to carbachol ad isoprenaline, also known as the cardiac chronotropic sympathetic dominance. Groups are normoxia (N, open bar or symbols, *n* = 9) hypoxia (H, gray bar or symbols, *n* = 10), normoxia with miR‐21‐5p (NA, blue bar or symbols, *n* = 8), and hypoxia with miR‐21‐5p (HA, red bar or symbols, *n* = 8). Statistical differences are (*p* < 0.05), *Two‐way ANOVA with FDR post hoc comparison.

Further, using Langendorff preparations of the isolated chicken embryo heart, we show that hypoxic incubation reduced the left ventricular contractility index (CI) and elevated left ventricular end diastolic pressure (LVEDP) and the ventricular relaxation index *Tau* (Figure 6 a‐d). In addition, hypoxic incubation blunted the embryonic cardiac chronotropic response to the muscarinic receptor agonist carbachol while tending to enhance the embryonic heart rate response to the β_1_ adrenergic receptor agonist isoprenaline (Figure 6e & 6f). This translated to a significant increase in the ratio of the maximal heart rate response to each agonist, also known as the cardiac sympathetic dominance of autonomic regulation in hypoxic chicken embryos (Figure 6g). Treatment with synthetic miR‐21‐5p (agomiR) of hypoxic incubations from Day 13 to 19 prevented the cardiac systolic and diastolic dysfunction and the increase in cardiac sympathetic dominance (Figure 6a–f).

## 4 Discussion

Fetal hypoxia increases the cardiovascular risk in offspring, contributing to a significant burden on lifelong health. Here, we introduce a new therapeutic approach to address this problem that exploits epigenetic strategies by delineating a mechanistic pathway by which miR‐21‐5p modulates the cardiomyocyte hypoxia response. In this study, hypoxia activated a cardiomyocyte injury program through coordinated transcriptional and post‐transcriptional regulation, which was modulated by miR‐21‐5p. In neonatal rat cardiomyocytes, miR‐21‐5p pretreatment suppressed a subset of hypoxia‐responsive transcripts, altered transcript isoform usage (notably Sfpq and Lgr4), attenuated apoptotic signaling, and shifted gene networks toward improved Ca^2+^ handling, β‐adrenergic and PI3K‐related pathways. At the systemic level, *in ovo* administration during developmental hypoxia prevented asymmetric growth, restored systolic and diastolic performance, and normalized autonomic responsiveness in chicken embryos. Together, these data indicate that miR‐21‐5p exerts post‐transcriptional control linking hypoxia sensing to effectors of cardiomyocyte survival and function.

### 4.1 Hypoxia‐Induced Transcriptional Responses in Neonatal Cardiomyocytes and the Effect of miR‐21

In this context, the data in this study show transcriptional changes in neonatal cardiomyocytes consistent with established hypoxia responses, characterized by enrichment in adrenergic signaling and pathways involved in calcium homeostasis and apoptosis (Giussani 2016; Itani et al. 2018; Giussani 2021). Validation using external transcriptional datasets from in vivo mouse (GSE114532) and chicken (GSE12675) models of exposure to hypoxia during early development shows similar upregulation of stress response and pro‐apoptotic genes and disruption of calcium handling. In primary NRC exposed to hypoxia in vitro, we show that miR‐21‐5p pretreatment improved cell viability, an effect that may result from the repression of pro‐apoptotic (Cheng et al. 2009) and/or activation of cell survival (Yang et al. 2014) signaling. In addition, miR‐21‐5p pretreatment attenuated the transcriptional response elicited by hypoxia, reduced the number of hypoxia‐responsive differentially expressed genes, and shifted the expression program toward cardioprotective pathways, antiapoptotic regulation, and immune modulation. This is consistent with recent studies reporting that miR‐21‐5p plays a key role in improving cell survival by regulating similar responses in diverse tissues (Yang et al. 2018; Fang et al. 2022; Ji and Wang 2024; Cong et al. 2025).

Through intersection analysis, we dissected the complex interplay between miR‐21‐5p and hypoxia, revealing three distinct transcriptional programs. This analysis suggested a counteracting effect, characterized by enrichment in cardiac function and development pathways, suggesting that miR‐21‐5p directly protects cardiac function. Second, a persistent effect of miR‐21‐5p, occurring either under normoxia or hypoxia, enriched in immune‐related biological processes that may help to prevent later myocardial dysfunction (Qiu et al. 2025). A persistent effect of hypoxia, independent of the presence of miR‐21‐5p, is primarily associated with metabolic reprogramming, findings aligned with the well‐known metabolic effects of hypoxia in the heart (Li et al. 2025). In complement with this, we identified 28 DEGs as potential miR‐21‐5p targets. Crucially, 23 of these were differentially regulated between untreated and miR‐21‐5p‐treated hypoxic NRC, suggesting direct suppression by the miRNA. Network and pathway analyses implicated these targets in KEGG pathways altered by both hypoxia and miR‐21‐5p, supporting their role in mediating cardiac protection (Dai et al. 2020). Notably, several genes with differential exon usage and expression contained binding sites complementary to miR‐21‐5p, providing further evidence of the interaction of this agomiR with crucial pathways regulating cardiac physiology during development.

### 4.2 In Vivo Effects of miR‐21 Improving Cardiac Function in Chronic Hypoxia During Development

The hypothesis that miR‐21 upregulation during developmental hypoxia is cardioprotective was further tested by extending these initial molecular studies to physiological experiments in the chicken embryo. The chicken embryo is useful to isolate any direct effects of developmental hypoxia to the conceptus, independent of any confounding effects on the maternal and/or placental physiology (Mulder et al. 2002; Itani et al. 2016, 2018; Skeffington et al. 2018; Skeffington et al. 2020; Krause et al. 2024). Chicken embryos in late incubation respond to chronic hypoxia in a similar fashion to the late gestation mammalian fetus. Chronic hypoxia *in ovo* promotes a brain‐sparing effect, asymmetric growth restriction, endothelial dysfunction, and cardiovascular sympathetic dominance in the late‐incubation chicken embryo (Mulder et al. 2002; Itani et al. 2016, 2018; Skeffington et al. 2018, 2020; Krause et al. 2024). Further, chronic hypoxia *in ovo* programs persistent cardiac and endothelial dysfunction, ventricular hypertrophy, and hypertension in the adult bird (Skeffington et al. 2018, 2020). Accordingly, we showed that *in ovo* treatment of miR‐21‐5p during hypoxic incubation restored both inotropy and lusitropy, improving cardiac contractility as well as relaxation. This is consistent with better Ca^2+^ handling, and prior studies have linked exosomal miR‐21‐5p to increased SERCA2a and L‐type Ca^2+^ channel expression, improving contractility via PI3K signaling (Mayourian et al. 2018). The identification of Sfpq and Lgr4 as DTU candidates highlights potential novel post‐transcriptional modulators of Ca^2+^ homeostasis needing functional validation. Supporting evidence includes studies where hMSC‐derived or patient‐derived exosomes enriched for miR‐21‐5p improved contractility, reduced infarct size and fibrosis, and enhanced calcium‐handling gene expression (Mayourian et al. 2018; Qiao et al. 2019). Chronic fetal hypoxia also up‐regulates cardiac β‐adrenergic receptor (βAR) signaling (Lindgren and Altimiras 2009; Niu et al. 2018; Allison et al. 2020). This is a well described compensatory response to the weakened contractile function, in order to maintain cardiac output in the fetus exposed to developmental hypoxia (Giussani et al. 2012). However, increased myocardial contractility due to heightened sympathetic excitation and diminished parasympathetic reactivity are unsustainable, and are therefore strongly predictive of cardiovascular disease and eventual heart failure in humans (Bristow 2000; Danson et al. 2009). Further evidence correlating these functional changes with structural data is required. Indeed, previous studies have demonstrated how *in ovo* treatments lead to improved cardiac function in chronic hypoxic embryos occur along with a reversion of the hypoxia‐induced cardiac remodeling (Itani et al. 2020). Nonetheless, this study reports cardiac functional data showing that the altered cardiac chronotropic responsiveness to autonomic receptor agonists signifying sympathetic dominance was prevented in hearts of chicken of hypoxic chicken embryos treated with miR‐21‐5p.

### 4.3 Translational Perspectives of miR‐21 Into Human Pregnancy Affected by FGR

Previous work has suggested that miRNA expression profiles in the human placenta may be linked with the regulation of fetal growth. As the function of the placenta is to promote fetal growth through its own proliferation and invasion into the maternal decidua, downregulation of the proliferative and migration effects of miR‐21 in the placenta has been linked to poor fetal growth and low birth weight (Maccani et al. 2011; Kochhar et al. 2022). This is interesting as low birth weight is a powerful predictor of risk for disease later in life, including coronary heart disease. We show that *in ovo* treatment of miR‐21‐5p during hypoxic incubation restored asymmetric growth in the hypoxic chicken embryo. Furthermore, miR‐21‐5p treatment reverted the detrimental effects of chronic hypoxia on fetal growth (Giussani et al. 2007), improving the use of nutritional resources as suggested growth efficiency and partition of resource indexes. Therefore, these data highlight direct protective effects of miR‐21‐5p on fetal growth that transcend its proliferative effects at the level of the mammalian placenta, opening new avenues of research into the direct effects of miRNAs in the regulation of fetal growth in healthy and complicated pregnancy.

In summary, miR‐21‐5p is a potent modulator of growth and cardiovascular function during developmental hypoxia. It improves cell survival, reprograms hypoxia‐responsive transcriptional networks toward adaptive pathways, protects against growth restriction, and enhances cardiac function during chronic developmental hypoxia. Key mechanistic links involve HIF‐regulated transcriptional responses, β‐adrenergic receptor (βAR) and PI3K signaling, modulation of calcium‐handling genes, and a set of direct and post‐transcriptionally regulated targets. This work supports the significant translational potential of changes in the expression of miRNA‐21 not only as biomarkers of, but also as a therapeutic target to protect against low birth weight and developmental origins of cardiovascular disease in offspring of complicated pregnancy.

## Author Contributions

Conceptualization: Bernardo J. Krause, Dino A. Giussani. Methodology: Bernardo J. Krause, Ximena Calle, Ranya V. Kumar, Youguo Niu, Oriana Ramirez‐Herrera, Sage G. Ford, Rachael C. Crew, Anna L.K. Cochrane, German A. Arenas, Alex Di Genova. Data and Statistical Analysis: Bernardo J. Krause, Ranya V. Kumar, Youguo Niu, Damariz Gonzalez, Barbara Bernal, Anna L.K. Cochrane, German A. Arenas, Alex Di Genova. Writing – Original Draft: Bernardo J. Krause, Ximena Calle, Youguo Niu, German A. Arenas, Alex Di Genova, Dino A. Giussani. Writing – Review and Editing: Bernardo J. Krause, German A. Arenas, Alex Di Genova, Dino A. Giussani. Visualization: Bernardo J. Krause, Ranya V. Kumar, Damariz Gonzalez, Anna L.K. Cochrane, Alex Di Genova. Supervision: Bernardo J. Krause, Alex Di Genova, Dino A. Giussani. Funding Acquisition: Bernardo J. Krause, Dino A. Giussani, Alex Di Genova, Dino A. Giussani. Funding acquisition: Bernardo J. Krause; Dino A. Giussani.

## Funding

The British Heart Foundation (PG/10/99/28656; Dino A. Giussani). Fondecyt Regular 1220421 (Bernardo J. Krause). Fondecyt Postdoctorado ANID 3240620 (Ximena Calle).

## Conflicts of Interest

The authors declare no conflicts of interest.

## Supporting information


**Figure S1:** Transcriptional effects of fetal chronic hypoxia on mice and chicken heart. Volcano plots showing DEG relative to normoxia in cardiac tissue from mice fetuses (a) and chicken embryos (d) exposed to hypoxia. UpSet plots and polar plot displaying Kegg pathways enriched in cardiac tissue from mice fetuses (b, c) and chicken embryos (e, h) exposed to hypoxia. Venn diagrams of enriched KEGG (f) and gene‐ontology biological processes (g) enriched in the transcriptional datasets analyzed (GSE114532 and GSE12675) and neonatal rat cardiomyocytes exposed to hypoxia (Hx NRC).

**Figure S2:** miRNA‐21‐5p has a mild‐effect on the transcriptional profile in cardiomyocytes under normoxia. (a) Volcano plots showing DEG relative to normoxia in NRC treated with miR‐21‐5p. (b) UpSet plot showing the overlap among enriched genes (horizontal bars represent the number of genes in each gene set, while the vertical bars show the size of the between sets, and connected dots below the vertical bars indicate which gene sets are included in each intersection in NRC exposed to normoxia in the presence of miR‐21‐5p). No enriched KEGG pathways were found.

## Data Availability

All data generated or analyzed during this study are included in this published article. Additional data are available from the corresponding author upon reasonable request.
