# Supplementary figures and images for: MiR‐21‐5p Protects Embryonic Growth and Heart Function During Developmental Hypoxia by Dampening HIF Responses and Altering Gene Expression
Source: Compr Physiol. 2026 May 15;16:e70173. doi: 10.1002/cph4.70173 (PMC13178401; doi:10.1002/cph4.70173)

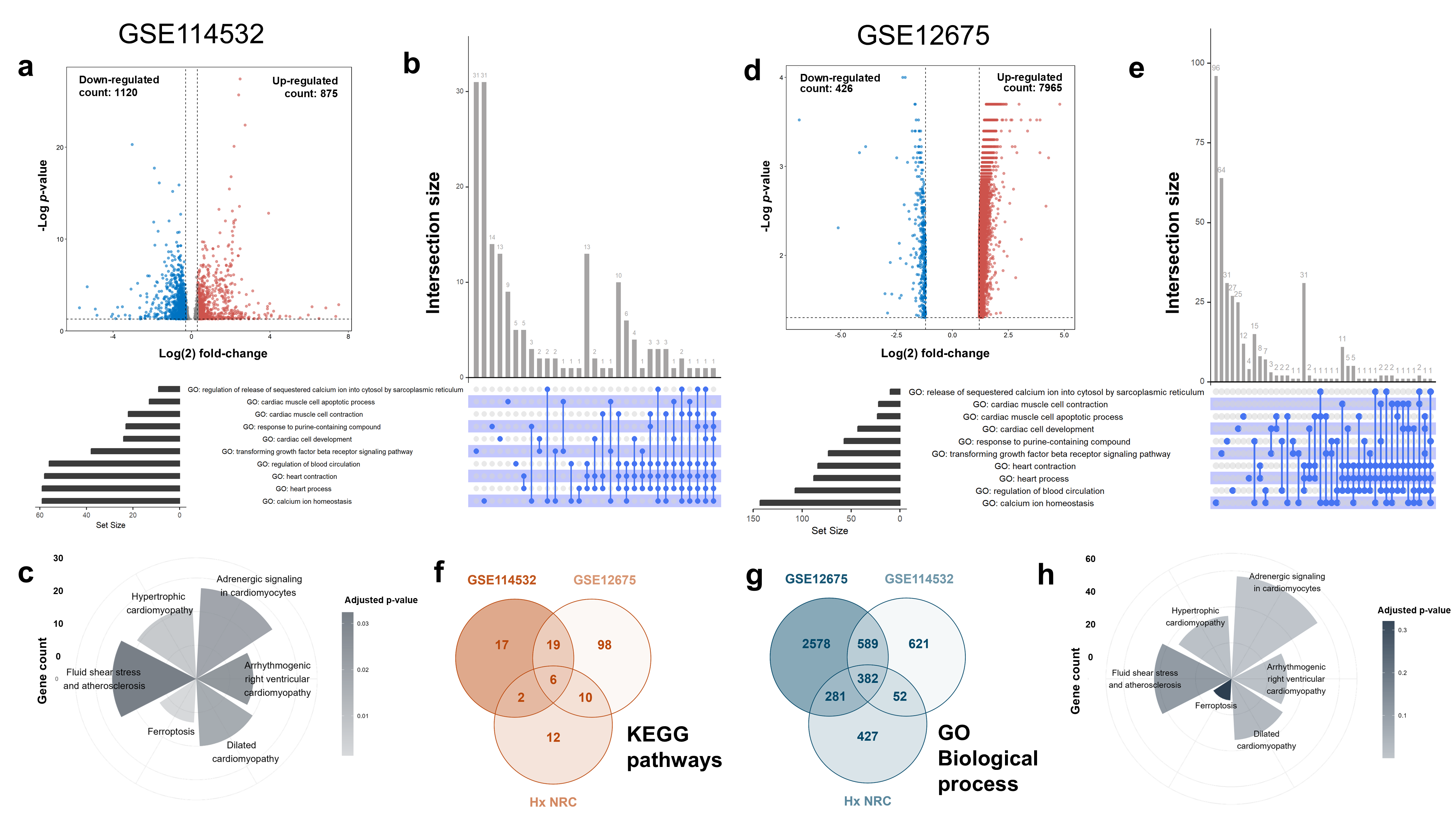

Supplement: Supplementary file 1 — Figure S1: Transcriptional effects of fetal chronic hypoxia on mice and chicken heart. Volcano plots showing DEG relative to normoxia in cardiac tissue from mice fetuses (a) and chicken embryos (d) exposed to hypoxia. UpSet plots and polar plot displaying Kegg pathways enriched in cardiac tissue from mice fetuses (b, c) and chicken embryos (e, h) exposed to hypoxia. Venn diagrams of enriched KEGG (f) and gene‐ontology biological processes (g) enriched in the transcriptional datasets analyzed (GSE114532 and GSE12675) and neonatal rat cardiomyocytes exposed to hypoxia (Hx NRC). [file CPH4-16-e70173-s002.tif]

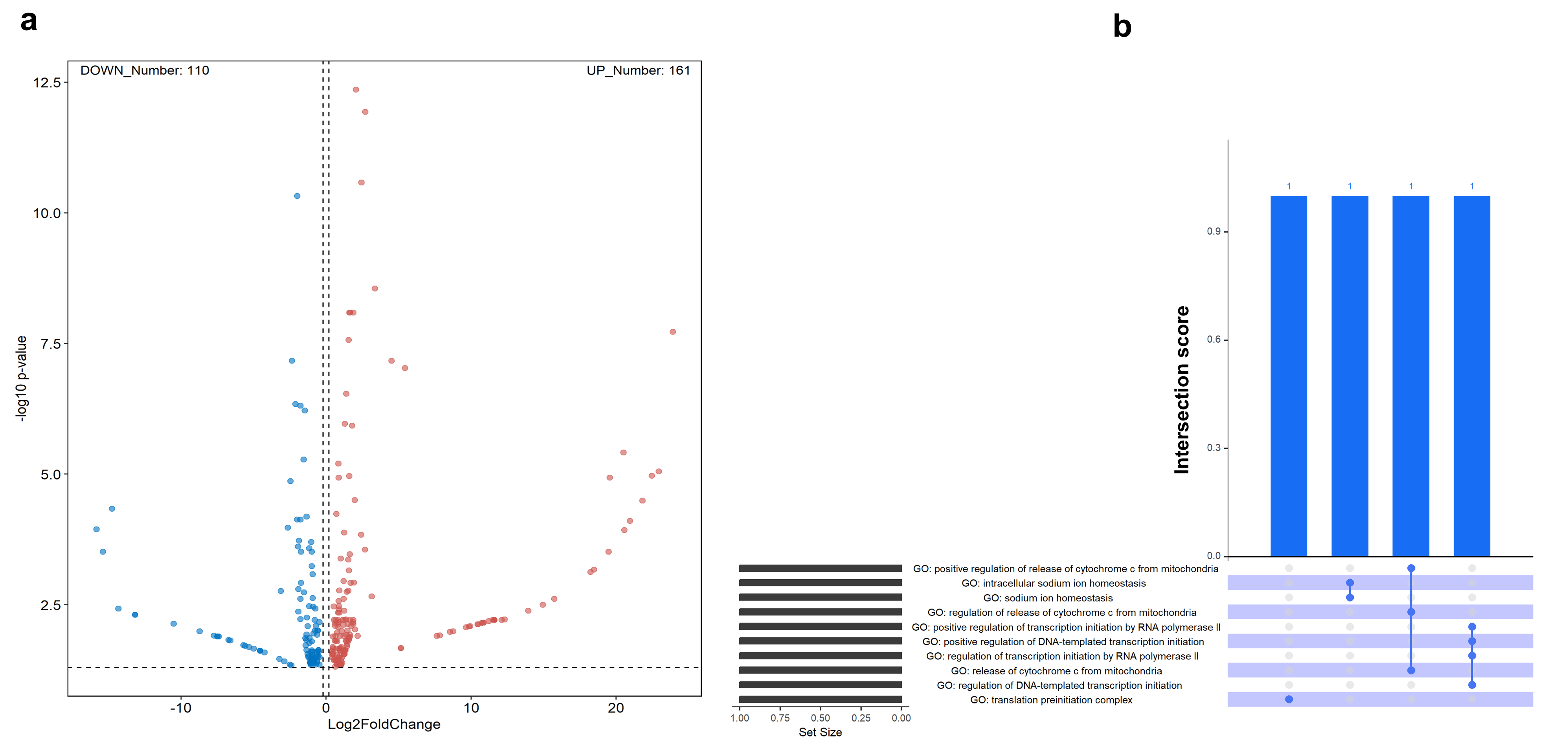

Supplement: Supplementary file 2 — Figure S2: miRNA‐21‐5p has a mild‐effect on the transcriptional profile in cardiomyocytes under normoxia. (a) Volcano plots showing DEG relative to normoxia in NRC treated with miR‐21‐5p. (b) UpSet plot showing the overlap among enriched genes (horizontal bars represent the number of genes in each gene set, while the vertical bars show the size of the between sets, and connected dots below the vertical bars indicate which gene sets are included in each intersection in NRC exposed to normoxia in the presence of miR‐21‐5p). No enriched KEGG pathways were found. [file CPH4-16-e70173-s001.tif]
